# Making Mathematical Research Data FAIR: Pathways to Improved Data Sharing

**DOI:** 10.1038/s41597-024-03480-0

**Published:** 2024-06-22

**Authors:** Tim O. F. Conrad, Eloi Ferrer, Daniel Mietchen, Larissa Pusch, Johannes Stegmüller, Moritz Schubotz

**Affiliations:** 1 Zuse Institute Berlin, Berlin, Germany; 2 FIZ Karlsruhe - Leibniz Institute for Information Infrastructure, Berlin, Germany; 3 Ronin Institute for Independent Scholarship, Montclair, USA

**Keywords:** Scientific community, Social sciences

## Abstract

The sharing and citation of research data is becoming increasingly recognized as an essential building block in scientific research across various fields and disciplines. Sharing research data allows other researchers to reproduce results, replicate findings, and build on them. Ultimately, this will foster faster cycles in knowledge generation. Some disciplines, such as astronomy or bioinformatics, already have a long history of sharing data; many others do not. The current landscape of available systems for sharing research data is diverse. In this article, we conduct a detailed analysis of existing web-based systems, specifically focusing on mathematical research data.

## Introduction

The importance of sharing and citing research data is steadily gaining recognition as a foundational element in scientific research across different fields and subjects. Sharing research data allows other researchers to reproduce results and replicate findings^1,2^. Ultimately, this promotes the generation of knowledge at a faster pace. Some disciplines already have a long history of sharing data and are benefiting from it^3^, but many others do not.

Although the term *data sharing* is not used unambiguously in the literature^4^, technically, data sharing is mainly organized through Research Data Repositories, which can be roughly classified into four categories: institutional, disciplinary, multidisciplinary, and project-specific^5^. Further, the terms *repository*, *platform* and *portal* are often used interchangeably, yet they usually serve different purposes. In our context, a repository is defined as a digital system mainly for the long-term storage and dissemination of research data - usually with some kind of (web) user interface. A portal, in contrast, is a web-based interface that aggregates and facilitates the discovery of research resources from different origins. Platforms, on the other hand, refer to extensive systems offering a variety of functionalities beyond basic data storage, encompassing collaboration tools and analytical capabilities. In cases where we do not distinguish between the three, we use the term open data *system*.

In the field of mathematics, significant progress has already been made in terms of research data sharing over the last 15 years. Since then, multiple new repositories have been established for sharing data types, such as theorem libraries or number sequences (see Table 1 for more examples). As a highly structured and rigorous field, mathematics fits well with the development of shared data resources. Particularly, theorems and proofs can be conveniently disseminated and checked using available checking engines^6,7^. Overall, the available sites and repositories provide mathematicians with a large assortment of mathematical objects that can be utilized to solve new problems, establish new theories, and increase knowledge - not only in mathematics.

**Table 1:** Overview of key data types in mathematical research.

Category	Types	Description
Symbolic data	Formulae, Theorems, Proofs, Functions	Consist of expressions and notations for abstract reasoning and the articulation of mathematical concepts.
Numeric data	(Integer) number sequences, Matrices, Tensors, Finite lattices	Comprise numerical values and organized structures essential for a broad range of mathematical applications, including analytical processes, representational tasks, and computational challenges.
Geometric data	Curves, Surfaces, High-dimensional objects, Polytopes	Involve the mathematical and algorithmic depiction of shapes and structures, pivotal in the fields of geometry, topology, and related disciplines.
Models	Math models, BioModels	Represent simplified versions of real-world phenomena designed for predictive analysis and hypothesis testing.
Observational data	Simulations, Experiments, Observations	Encompass data gathered from direct observations, experiments, and simulations to explore and verify natural phenomena.
Text data	Research papers, encyclopedia entries	Include texts such as scholarly articles and academic books, which serve as primary sources for mathematical research and discussion.

It should be noted that particular types of research data have traditionally been shared as part of scholarly publications. However, research data repositories and scholarly publications serve complementary but distinct roles in mathematical research. Scholarly publications mainly articulate theoretical advancements, methodologies, and analytical insights, whereas research data repositories can provide various other types of data, for example, empirical datasets underpinning these academic discussions. This distinction emphasizes the importance of research data repositories in enabling empirical scrutiny and verification, a fundamental aspect of the scientific process. Thus, the integration of both research outputs enhances the robustness and credibility of mathematical research, highlighting the essential role of data repositories alongside traditional scholarly narratives.

However, despite the advancements made in the mathematics community, data sharing is still a subject that requires continuous attention and improvement. Researchers, institutions, and funding agencies must prioritize the development of rules and infrastructure that facilitate the sharing and citation of research data to increase the prevalence of good data-sharing practices in mathematics - and other fields. By doing so, we may develop an environment for research that is more open and collaborative, thereby accelerating the rate of scientific discovery.

Also in other fields, data sharing enables more transparent and repeatable scientific research, making it an increasingly important topic in recent years. By releasing data openly, researchers can increase the likelihood that their findings can be repeated and validated by others, thus bolstering the credibility of the results. However, issues persist in ensuring that data is shared in an efficient and accountable manner. To ensure its integrity and usability, the shared data must be carefully curated and documented. Caution needs also to be taken such that privacy and confidentiality issues are addressed to prevent misuse or misinterpretations of the data. By establishing best practices for data sharing and citation and facilitating the creation of standardized metadata and data management standards, these challenges can be overcome.

In conclusion, the sharing and citation of research data is a crucial part of scientific research that has the ability to boost collaboration and speed the production of new knowledge. The field of mathematics has made substantial progress in this area, but additional efforts are required to guarantee that data sharing becomes a generally accepted and well-supported practice in all fields.

### Objectives & Outline

In this paper, we aimed to address the following research questions, with a particular focus on mathematical research data.

1. What is the current status of open data systems (i.e. repositories, portals or platforms) in academia?
2. What are the main requirements for an open data system?
3. What are the biggest challenges and obstacles that are preventing the successful implementation of widely used open data systems?

We have structured the paper as follows: We first give the necessary background and emphasize the significance of open data systems in mathematical research and their role in promoting Open Science in the following section. In the following methods section, we describe our methodology for compiling and evaluating a comprehensive list of mathematics-related open data systems. The open data systems that made it to the final list are described in the results section, in which we provide a comprehensive analysis, assessing their features and conformance to the FAIR principles. The discussion section provides an analysis of the results, highlighting the challenges and limitations of the existing open data systems.

## Open Science in Mathematics: The Role of Open Data and Data Systems

The Open Science movement advocates for accessible scientific research to foster collaboration and transparency^8^. It emphasizes open access to publications, software, data, and educational materials, aiming to enhance research application and efficiency. For instance, the global response to the COVID-19 pandemic serves as a compelling example of Open Science’s significance. Researchers worldwide quickly shared genomic sequences of the virus, clinical data, and research findings through open-access platforms, significantly speeding up the understanding of the virus and the development of vaccines and treatments. Ultimately, this openness goes beyond the data itself and additionally allows other researchers to access and replicate the computational environment (if needed) and used methods or algorithms.

The FAIR principles, on the other hand, specifically focus on data management and (machine-friendly) accessibility aspects with a focus on meta-data, rather than the broader transparency of research methodologies or the sharing of computational tools and software. Both, Open Science and the FAIR principles are beneficial in their own ways, but the real benefits accrue when they are combined.

In mathematics, open data is particularly crucial, enabling replication, validation, and new research avenues. The main goal is to make the scientific community more transparent and efficient by making it significantly easier to re-use previous research results. This openness not only facilitates collaboration within the mathematical community but also enhances cross-fertilization with other fields, fostering interdisciplinary approaches that can lead to new discoveries and innovations. This section examines the impact of open data in mathematics and the role of digital systems in broadening its accessibility.

### Open Data in Mathematics

As a central component of Open Science, open data refers to the practice of making research data publicly accessible under open licenses. This practice facilitates the replication and validation of findings by allowing other researchers to verify and expand upon previous research. In addition, open data enables the investigation of new research questions and hypotheses, as well as the combination of data from multiple sources to uncover novel insights and patterns. As a result, open data is becoming the norm in an increasing number of scientific disciplines.

The field of mathematical research provides an intriguing example in this regard. Numerous data types, including symbolic formulae and theorems, numerical arrays, and observational information, characterize mathematical research (see Table 1 for an overview). Understanding these various data types is essential for analyzing and communicating mathematical research effectively.

With a focus on mathematical research data, we will investigate open data systems and how they can be utilized to make such data accessible to the general public. By understanding the advantages and disadvantages of open data, researchers can make well-informed decisions regarding how to share their research and contribute to the expanding Open Science movement.

### Open Data Systems

In recent years, data sharing has become an essential component of scientific research, as it enables researchers to increase the impact of their work and promote transparency and collaboration. Open data systems (repositories, portals or platforms) are digital environments where scientists can store, exchange, and access datasets, sometimes even offering advanced functionality, such as data analysis. Usually, these systems include data management tools, metadata standardization, and version control. Zenodo and Figshare (although having a commercial background) stand out as prominent representatives highly relevant also to the mathematics community, since they support a wide range of data formats and allow for the efficient organization, sharing, and preservation of mathematical research data.

Nonetheless, the process of data sharing poses various challenges, such as ensuring open access and adhering to the *Accessibility* criterion of the FAIR principles. In this context, *open access* refers to the removal of financial, legal, and technical barriers to data, making it freely available to anyone. On the other hand, *Accessibility* under the FAIR principles refers to the data being retrievable by both humans and machines under well-defined conditions, ensuring that once data is accessed, it remains available and usable. In this regard, an open data system serves as a centralized repository for storing and sharing research data, thereby offering a solution to these challenges by providing features that support both open access and FAIR principles. Such a system enhances data sharing and reuse through easy-to-navigate access, enhanced discoverability, simplified data submission mechanisms, robust metadata management functionalities, and conformance with FAIR principles.

In recent years, the criteria for evaluating open data system have been shaped by both community standards and scholarly research, emphasizing the importance of accessibility, usability, and compliance with established data principles. From this body of literature, we have selected the following key features for open data systems:

1. Free use: The open data system should be free to use for researchers, allowing them to share, access, and reuse data without financial barriers. This encompasses the provision of open licenses that facilitate free reuse and redistribution, aligning with the principles of open access and open science. This is supported by the Budapest Open Access Initiative and reinforced by the principles of the Berlin Declaration on Open Access to Knowledge in the Sciences and Humanities, which advocate for the free availability and reuse of scholarly materials^9,10^.
2. Accessibility: Researchers should be able to access the data from any location and computational environment, via a standard web browser. Easy accessibility promotes transparency, facilitates reproducibility, and helps to avoid duplication of efforts. This concept is grounded in the belief that scientific knowledge should be accessible to all, a principle articulated in declarations such as the UNESCO Recommendation on Open Science^11^.
3. Data submission mechanisms: Researchers should be able to submit data to the repository, making it available for future use and replication. This feature promotes the sharing of data and transparency of research results and aligns with best practices in data management and sharing, as outlined for example by the European Commission^12^ or the Research Data Alliance on the example of COVID-19 data^13^.

Moreover, an effective open system should aim to fulfill additional features such as:

FAIR principles compliance: The system should adhere to the FAIR principles^14^, which emphasize the importance of data being Findable, Accessible, Interoperable, and Reusable. These principles place particular importance on the ability to process data by machines and have become a global standard for data management and sharing, endorsed by funders, publishers, and research institutions.
Data quality: The system should ensure that the data and meta-data is of high quality, reliable, and accurate, focusing on completeness, consistency, accuracy, and relevance to the respective domain. It should enable precise measurement and validation techniques. Regular evaluations, leveraging domain-specific standards and user feedback, should be conducted to ensure data integrity and relevance. This feature is critical to ensure that research data is useful and impactful for future research. Emphasis on data quality is grounded in the literature on data curation and quality assessment, such as the work by Heinrich *et al*.^15^, which discusses the dimensions of data quality and demonstrates its applicability and efficacy.
Metadata management: The system should provide tools for metadata management, including descriptions of the data, authors, and institutions. Metadata helps researchers to discover, access, and understand the data. The significance of metadata management is supported by studies like those by Leipzig and colleagues^16^, which highlight the role of metadata in enabling data discovery, access, understanding, and reproducibility of results.
(Meta)data format and standardization: The data system should support various data formats and adhere to standardization guidelines to ensure the data is easily findable, accessible, interoperable, and reusable. The need for standardization to ensure interoperability and reusability of data is outlined in initiatives such as the Data Documentation Initiative (DDI)^17^, the Wellcome Trust foundation^18^ and standards like ISO/IEC 11179.
Security and privacy: The system should ensure the security and privacy of the data, protecting sensitive information from unauthorized access and misuse. The importance of data security and privacy protections is well-documented in data governance literature and guidelines, such as those by the National Institutes of Standards and Technology (NIST).
User-friendly interface: The system should be easy to use and accessible, with low entry barriers, enabling scientists from diverse backgrounds to participate in the Open Science movement. This feature includes clear and concise documentation, as well as intuitive interfaces to upload and retrieve data. The requirement for user-friendly interfaces is supported by various usability studies, which emphasize the importance of low entry barriers and intuitive navigation for fostering broader engagement with open data^19,20^.

In the following, we will describe and review existing research data systems along those features (see Results Section). Keep in mind that in this work, however, we will restrict our focus on systems that contain significant mathematical research data. Before we dive into these details, we first introduce how the data was collected.

## Methods

To assess the current status of open data systems in the field of mathematics, the first challenging step consisted of obtaining a list of all relevant systems in the field, operational at the time of writing this article. A systematic search based solely on an examination of publications is not a feasible approach in this case, since most open systems exist without being explicitly documented in the technical literature. Instead, most of the current systems are only findable through search engines. Thus, our approach to getting an overview of the current ecosystem had to combine a literature review with the direct results obtained in a search engine. We have selected Google Scholar as our primary search engine since it is widely recognized for its comprehensive index of scholarly articles, theses, books, and conference papers. Given our focus on identifying open data systems documented within the academic community, Google Scholar provided a direct route to relevant, peer-reviewed publications in the field of mathematics, which is an advantage over sources such as Wikipedia or Wikidata. We also used the *zbMATH Open* system as an alternative index but did not find relevant results that have not been already covered by Google Scholar.

We started our search by analyzing published articles found in Google Scholar obtained by combining the terms “mathematics”, “research data”, “scientific data”, “research metadata”, “scientific metadata”, “portal”, “repository”, “infrastructure”, “platform”, “metadata management” and “FAIR”. By examining these publications as well as the URLs mentioned in them, we obtained a first tentative list of open systems, not only in the field of mathematics. We also identified further publicly-accessible portals directly through search engines using the same keywords and through searches in aggregators of data repositories that included FAIRsharing^21^, MathHub^22^, OpenDOAR and re3data^23^. The list was finally completed based on the authors’ knowledge. The initial search was performed during the period June 2022 – October 2022. A second round of searches took place in the period March 2023 – June 2023. It is also important to note that as a restriction on all inquiries, the availability of English-language content was mandated.

From the initial list of systems, we excluded those that did not meet the essential requirements outlined in section on Open Data Systems. Specifically, we discarded systems that were not free to use, not publicly accessible, or did not offer the option of data submission. This means that we excluded systems that did not allow researchers, irrespective of their institutional affiliation or geographical location, to submit their data freely. For this study, this forced us to exclude known resources in the mathematical community such as the Encyclopedia of Triangle Centers^24^, the ISGCI (Information System on Graph Classes and their Inclusions)^25^, the Graded Ring Database^26^, the L-functions and modular forms database^27^ as well as a long list of publicly-accessible institutional repositories that only accept submissions by its members such as ATLAS of Finite Group Representations https://brauer.maths.qmul.ac.uk/Atlas/v3/or MIZAR http://mizar.org/library/.

Platforms that just aggregate metadata and thus do not offer a mechanism to directly incorporate data and metadata submitted by users were also excluded from the study. These include aggregators such as re3data, DataCite^28^ and Dimensions.ai^29^. We also excluded open systems that did not include a significant amount of mathematical research data at the time of writing and were thus not relevant for our purposes. Among these systems were B2share^30^, Dryad^31^, Fairdomhub^32^, Mendeley Data^33^ and Vivli^34^.

Finally, within the mathematical ecosystem, there exists a distinct category of portals that mainly contain written articles that describe concepts within specific mathematical disciplines. These systems, often built on the MediaWiki framework, encourage user contributions through a wiki-based approach. However, they are often predominantly populated only by a small group of active contributors within the field. While these systems are free to use, accessible, and allow user contributions, they often display limited adherence to the FAIR principles. Notably, they frequently lack unique persistent identifiers for published articles, do not provide access to comprehensive metadata via an API, and often lack explicit license information. Due to these limitations, we chose not to include them in this work, as their inclusion would have significantly expanded the list with numerous items that only marginally comply with the FAIR principles. Nevertheless, given their significance in the field of open-access mathematical research data, we have included a non-exhaustive list of such systems in Section Wiki-based Math Data Sharing.

The final list of systems is included in Table 3. Each system has been evaluated based on Austian *et al*.^35^ using publicly available information, in the following categories (see Table 2): Infrastructure, Preservation, Security / Privacy, Archiving, Submission, Access / Sharing, Policy, and whether they are compliant with the FAIR principles^14^. This allowed us to identify presently implemented standards and features related to the management and sharing of research data, with a focus on mathematics. An analysis of the FAIR compliance for all systems is summarized in Table 5. Here, we evaluate the FAIR compliance by focusing on detailed criteria under each of the FAIR principles. For each system, we assessed:

- Findability (F1, F2, F3, F4): We verified the employment of globally unique and persistent identifiers for (meta-)data, the richness of meta-data that enhances discoverability, the explicit linkage between data and its meta-data through identifiers, and the registration or indexing of (meta-)data in searchable resources.
- Our assessment encompassed the mechanisms for retrieving (meta-)data via standardized, open, and universally implementable communication protocols. This included protocols’ support for necessary authentication and authorization procedures and ensuring meta-data remains accessible even if the data itself is no longer available.
- We examined the adoption of a formal, accessible, shared, and broadly applicable language for knowledge representation in (meta-)data. Additionally, we looked at the use of FAIR-compliant vocabularies and the inclusion of qualified references to other (meta-)data to support seamless integration with other datasets.
- This involved evaluating whether (meta-)data is accompanied by comprehensive and relevant attributes, clear and accessible data usage licenses, detailed provenance information, and whether it adheres to domain-relevant community standards.

**Table 2:** Evaluation criteria, based on Austian *et al*.^35^ and Wilkinson *et al*.^14^.

Category	Sub-category	Options
Infrastructure	systems	Dataverse ∣ Figshare ∣ MediaWiki ∣ Proprietary ∣ Open-Source (other)
	Cost	Free ∣ Free to access, but contribution needed for deposit
	Size	Size of repository (number of datasets)
Preservation	Redundancy	None ∣ Multiple redundant copies ∣ Geographically distributed redundant copies
	Persistent identifiers	No ID ∣ DOI ∣ other persistent ID ∣ non-persistent ID
	Persistent data deposit	None ∣ Long term data preservation
Security /	Security	None ∣ Authentication mechanism
Privacy	Privacy	None ∣ Distinction between public and private data
Archiving	Author identifier	None ∣ zbMATH Open Author ID ∣ ORCID ID ∣ SCOPUS ID ∣ Other
	Publication identifier	None ∣ zbMATH Open Document ID ∣ Reference to paper through DOI ∣ Other reference
	Time stamping	None ∣ Timestamp upon upload ∣ Timestamp for every version
Submission	Data types	No restriction ∣ Restrictions to specific types
	Data size	No restriction ∣ Restricted to maximal size
	Metadata	No metadata necessary ∣ Controlled Language ∣ Readme file
	Review/Data Quality	None ∣ Submissions are reviewed and approved for metadata and compliance
Access / Sharing	Online access	Data available for free and open download ∣ User registration needed
	API	None ∣ API for search available ∣ API for search and submission available
	License	CC0 ∣ Creative Commons License ∣ Other license (open) ∣ Other license (restrictive)
Policy	Mandate	No ∣ Yes (Info about: Under what authority does the repository operate (e.g. government)?)
	Data Ownership	No ∣ Yes (Info about: Who owns the ingested data?)
	Data Licensing	No ∣ Yes (Info about: How are the data licensed?)
	Preservation	No ∣ Yes (Info about: What is the practice for long-term preservation?)
	Succession plan	No ∣ Yes (Info about: What actions will be taken if the repository is closed?)
FAIR Principles	Findability	No ∣ Yes (Means: The data can be discovered by both humans and machines, for instance by exposing metadata and keywords to search engines)
	Accessibility	No ∣ Yes (Means: The (meta-)data is archived in long-term storage and can be made available using standard technical procedures)
	Interoperability	No ∣ Yes (Means: The data can be exchanged and used across different applications and systems)
	Reusability	No ∣ Yes (Means: The data is well documented and licensing information is provided

To ensure a thorough and accurate assessment, each criterion was verified through an exhaustive review of each portal’s documentation, available literature, and, when necessary, direct communication with the system providers. This approach enabled us to compile a detailed analysis of each portal’s alignment with FAIR principles, highlighting notable strengths and identifying key areas for enhancement.

The following section provides a brief description of each included systems, based on the evaluation criteria. Together, this information serves as the basis for the discussion on the presented research questions.

### Limitations of this Study

Despite our best efforts to compile a comprehensive list of open data systems in the field of mathematics, we recognize certain limitations inherent in the presented method. These limitations primarily stem from the scope of our search criteria, language restrictions, and the reliance on publicly available sources:

#### Search Criteria Limitations

Our approach was constrained by the specific keywords used. While these keywords were selected to cover a broad range of systems, we acknowledge that the very specialized and fragmented nature of the mathematics field may mean some niche or emerging systems that do not align closely with our chosen terms were inadvertently omitted.

#### Language Restrictions

The search was limited to English-language content and systems. This constraint likely resulted in the exclusion of valuable systems and repositories that operate in other languages, which may be particularly relevant in non-English speaking regions or international collaborations.

#### Reliance on Publicly Available Sources

Our methodology primarily relied on information that could be found through academic publications, search engines, and specific data repository aggregators. This approach may overlook systems that are not indexed in these sources or that prefer low-profile, community-specific engagement.

#### Influence of Authors’ Knowledge

The final list was also informed by the authors’ own knowledge and professional networks. While this allowed us to add systems not identified through the initial search, it also introduces a subjective element that could bias the results towards the authors’ specific areas of expertise.

In light of these limitations, it’s important to underscore that while our resulting list of open data systems is a robust and valuable resource, it is not exhaustive. We have endeavored to create a comprehensive directory, yet inherent constraints mean that it may not encompass every systems within the field.

## Results

This section examines the current state of Open Science and Open Data systems by analyzing the available literature and the implementation details of existing systems. The evaluation is based on the criteria presented in Table 2 and on the adherence to the FAIR principles. Apart from a short description of each system, summarized in Table 3, a comparison in terms of the most relevant features for data sharing has been included in Table 4.

**Table 3:** List of included portals, sorted alphabetically.

Portal Name	Math focus	Citability	License	# Items	URL
4TU Research Data	Multidisciplinary	DOI	CC0 (default), CC	>8300	data.4tu.nl
Archive of Formal Proofs	Proofs	URL	BSD, LGPL	~700	isa-afp.org
arXiv	Multidisciplinary	DOI, arXiv ID	CC, arXiv perpetual	>2, 2M	arxiv.org
Biomodels	Mathematical models	Model ID	CC0	>2500	ebi.ac.uk/biomodels
Database of Ring Theory	Rings	Internal ID	CC BY 4.0	~300	ringtheory.herokuapp.com
Encyclopedia of Graphs	Graphs	Graph ID	CC BY-NC-SA 3.0	>11M	atlas.gregas.eu
Figshare	Multidisciplinary	DOI	CC, MIT, GPL, Apache	>7, 3M	figshare.com
FindStat	Combinatorial statistics	Internal ID	CC BY 4.0	~2000	findstat.org
HAL	Multidisciplinary	DOI, idHAL	CC, copyright	>3M	hal.archives-ouvertes.fr
Harvard Dataverse	Multidisciplinary	DOI	CC0 (default), custom	>156000	dataverse.harvard.edu
Network Repository	Network datasets	URL	CC BY-SA	>6600	networkrepository.com
OEIS	Integer sequences	Internal ID	CC BY-NC 4.0	>350000	oeis.org
Open Science Framework	Multidisciplinary	DOI, Internal ID	diverse	>7M	osf.io
Open Science Library	Multidisciplinary	DOI	diverse	>100	codeocean.com
Papers with Code	Multidisciplinary	URL	CC BY-SA	>4000	math.paperswithcode.com
*π*-Base	Topological counterexamples	Internal ID	CC BY 4.0	~500	topology.pi-base.org
polyDB	Discrete geometric objects	Internal ID	undefined	~500M	polydb.org
Science Data Bank	Multidisciplinary	DOI, Internal ID	diverse	>7M	scidb.cn
SuiteSparse Matrix Collection	Sparse matrices	Internal ID	CC BY 4.0	~2800	sparse.tamu.edu
The House of Graphs	Graphs	Graph ID	Copyright	~22000	houseofgraphs.org
Wikidata	Multidisciplinary linked data	Wikidata ID	CC0	>100M	wikidata.org
Zenodo	Multidisciplinary	DOI, zenodo ID	diverse	>2, 8M	zenodo.org

**Table 4:** Main features of included portals.

Portal Name	Persistent ID	Author ID	Publication ID	Timestamping	API	Private data
4TU Research Data	✓ (DOI)	✓ (ORCID)	✓ (DOI)	✓✓	✗	✓
Archive of Formal Proofs	✗	✗	✓ (DOI)	✓	✗	✗
arXiv	✓ (DOI)	✓ (ORCID)	✓ (DOI)	✓✓	✓	✗
Biomodels	✓	✗	✓ (DOI)	✓✓	✓	✗
Database of Ring Theory	✓	✗	✗	✗	✗	✗
Encyclopedia of Graphs	✓	✗	✗	✗	✗	✗
Figshare	✓ (DOI)	✓ (ORCID)	✓ (DOI)	✓✓	✓✓	✓
FindStat	✓	✗	✓	✓✓	✓	✗
HAL	✓ (DOI)	✓ (ORCID)	✓ (DOI)	✓✓	✓✓	✗
Harvard Dataverse	✓ (DOI)	✓ (ORCID)	✓ (DOI)	✓✓	✓✓	✓
Network Repository	✗	✗	✗	✗	✗	✗
OEIS	✓	✓	✗	✗	✓	✗
Open Science Framework	✓ (DOI)	✓ (ORCID)	✓ (DOI)	✓✓	✓✓	✓
Open Science Library	✓ (DOI)	✗	✓ (DOI)	✓	✗	✓
Papers with Code	✓	✓ (ORCID)	✓ (DOI)	✗	✓✓	✗
*π*-Base	✓	✗	✓ (DOI)	✗	✗	✗
polyDB	✓	✗	✓ (DOI)	✗	✓	✗
Science Data Bank	✓ (DOI)	✓ (ORCID)	✓ (DOI)	✓✓	✓✓	✗
SuiteSparse Matrix Collection	✓	✗	✗	✗	✓	✗
The House of Graphs	✓	✗	✗	✗	✗	✗
Wikidata	✓	✓	✓ (DOI)	✓✓	✓✓	✗
Zenodo	✓ (DOI)	✓ (ORCID)	✓ (DOI)	✓✓	✓✓	✓

Persistent ID: ✗ No persistent ID for objects in the system, ✓ Objects are identified with a persistent ID, e.g. DOI

Author ID: ✗ No reference ID for referenced authors, ✓ Authors are referenced with an ID, e.g. ORCID

Publication ID: ✗ No reference ID for referenced publications, ✓ Publications are referenced with an ID, e.g. DOI

Timestamping: ✗ No timestamp, ✓ Timestamp upon upload, ✓✓ Timestamp for every version

API: ✗ No API, ✓ API for search, ✓✓ API for search and submission

Private data: ✗ No distinction between public and private data, ✓ Creation of private repositories or datasets is possible

### Open Data Systems

For each system listed, a brief description of its key features is provided, including information related to its creation, technological framework, objectives, and mathematical focus. If available, we include scientific papers and white-papers in which the systems are described. If no such paper is available, we refer directly to the website.

#### 4TU Research Data

4TU.ResearchData is an online data repository for science, engineering and design, managed by the 4TU.ResearchData Consortium. It aims to facilitate the sharing of research datasets and guarantees their long-term access by adhering to FAIR principles. The repository has been online since 2010^36^, it is based on Figshare technology, and it is hosted and managed by the TU Delft Library. As of June 2023, it hosts slightly more than 8,000 items, which include 7,850 datasets and 174 software items. The vast majority of the items belong to the field of atmospheric sciences and climate studies. About 100 items are assigned to mathematical categories, including computation theory, numerical and computational mathematics, mathematical physics, applied and pure mathematics. Every uploaded dataset receives a DOI and can be assigned a license, the most popular being CC0 and CC BY 4.0. One of the most distinct attributes of this repository is its advanced functionality for software preservation, including integration with GitHub and GitLab, dedicated licenses for software and a repository sandbox for testing. Currently, the FAIR principles regarding findability, accessibility and interoperability are fully fulfilled while those concerning reusability are only partially fulfilled.

##### Relevance of the repository to the mathematical research community

While having a focus on atmospheric sciences and climate studies, the repository offers long-term preservation and sharing of datasets and software also in a dedicated section for mathematics. Its advanced functionality for software preservation is especially crucial for computational mathematics, ensuring that mathematical software and algorithms remain accessible and usable over time.

#### Archive of Formal Proofs

The Archive of Formal Proofs^37,38^ is a collection of 700 proofs from various areas of mathematics, including number theory, algebra, analysis, and geometry, among others. All included items have passed both classical peer review and have been verified by the theorem prover Isabelle^39^. The repository additionally contains proof libraries and examples for Isabelle system. The site was launched in 2004 and is maintained by the Isabelle user community. The content is organized in the style of a journal, with each article being a set of Isabelle theories and proofs accompanied by definitions, theorems and corollaries which are written in the dedicated input language Isar, and as such executable using Isabelle. Each entry is citable via a locally unique identifier string. The proofs are available under BSD and LGPL software licenses. Overall, the Archive of Formal Proofs fulfills just over half of the FAIR Principles.

##### Relevance of the repository to the mathematical research community

This repository is especially relevant for mathematicians focusing on formal proofs and theorem verification, providing a trusted system for their dissemination.

#### arXiv

arXiv is an open access repository for scholarly preprints and postprints in eight subject areas, including mathematics, physics and computer science. It was founded in 1991, and it is currently maintained by Cornell University. Articles can be submitted to arXiv at no cost. Although submissions undergo a moderation process to ensure compliance with basic standards and field appropriateness, they are not subject to traditional peer review. As of June 2023, it is hosting well over 2 million scholarly articles organized into 32 distinct categories, with over 500,000 of them being within the field of mathematics. The repository has a strong commitment to open access, ensuring that all of its content is freely available to the public. It fully complies with the FAIR principles for findability, accessibility, and reusability while only partially fulfilling the principles for interoperability. Authors can choose from several license types under which an item is made available, including various CC-BY variants, CC0, and an arXiv specific license. Moreover, arXiv supports the inclusion of ancillary files such as tables, code or images, providing supplementary context or data supporting the research.

##### Relevance of the repository to the mathematical research community

ArXiv is indispensable to mathematicians globally as a primary open access repository. It provides an extensive collection of mathematical preprints and postprints, facilitating early dissemination and open discussion of research findings. However, it is crucial to note that scientific quality or validity is not evaluated by the provider.

#### Biomodels

The European Bioinformatics Institute (EBI) hosts the BioModels^40^ repository, an open data resource that provides access to more than 1000 curated computational models in systems biology. These models are derived from descriptions of biological phenomena found in the scientific literature, ranging from molecular and cellular processes to more complex views of whole organisms. Each model is assigned a unique and permanent identifier that can be used to cite the models. The repository supports interoperability by permitting models to be downloaded in various formats, such as SBML (Systems Biology Markup Language) or as an ODE system in the Octave syntax. BioModels provides a comprehensive and easily accessible compilation of mathematical models that describe biological systems, thereby promoting research, replication, and collaboration. All models are shared through the CC0 license.

##### Relevance of the repository to the mathematical research community

Though primarily focused on Systems Biology, BioModels is increasingly relevant to mathematicians interested in biological systems’ computational modeling.

#### Database of Ring Theory

The Database of Ring Theory is a comprehensive collection of rings and modules. It was created in 2013 by Ryan C. Schwiebert and currently holds a total of 162 rings, which can be explored through a list of 175 properties. It also stores data on 11 modules, classified according to 51 distinct properties and 63 theorems that are classified into 8 categories. Each object within the database can be accessed through its URL, which contains an internal ID. While the data within this portal is also published in a repository on GitHub^41^, it is important to note that the license information, specifically the CC BY 4.0 license, is not explicitly mentioned on the website. However, this database encourages user participation by enabling data submissions through pull requests on the associated GitHub repository. The current implementation of the database has limited compliance with the FAIR principles.

##### Relevance of the repository to the mathematical research community

This specialized database supports the mathematical research community by providing a comprehensive collection of ring theory-related data.

#### Encyclopedia of Graphs

The Encyclopedia of Graphs is an online repository of graph collections established in 2012 as part of the GreGAS project, funded by the European Science Foundation. As of 2023, it holds 46 collections that encompass not only graphs but also graph-like structures such as maps, configurations, and networks. Users can filter the objects in each collection using a list of over 30 distinct properties that vary depending on the specific collection. Each graph is assigned a unique Universal Graph Identifier, enabling direct access to its properties. The graph data within the repository is provided in the canonical sparse6 format and is released under the CC BY-NC-SA 3.0 license. The repository partially complies with the FAIR principles, but lacks interoperability due to the absence of machine-readable metadata, accessible through an API.

##### Relevance of the repository to the mathematical research community

The Encyclopedia of Graphs offers an invaluable resource for mathematicians interested in graph theory and its applications.

#### Figshare

Figshare is a general purpose scientific repository operated by commercial UK-based company Digital Science & Research Solutions Ltd. It was established in 2011 and supports researchers from all disciplines^42^. As of June 2023, it contains more than 7 million records, including around 350,000 entries for mathematics. The mathematical records consist mainly of figures, datasets, and journal articles. Figshare offers generation of DOI’s for hosted content, and various licenses are available, such as the various Creative Commons licenses, GPL variants, Apache and MIT licenses. Furthermore, Figshare offers a public REST-based API, OAI-PMH endpoints, and the possibility to integrate GitHub, GitLab, Overleaf and other applications. Figshare fulfills the FAIR principles for findability, accessibility and interoperability and partially fulfills the principles for reusability. For interoperability, Figshare supports OAI-PMH which enables the inclusion of qualified references in (meta-)data. The domains which define the topics of records use controlled vocabularies. A license can be selected for a record for reusability, but the selection is not required. Figshare supports a data citation metadata schema that can be customized by the users.

##### Relevance of the repository to the mathematical research community

With its substantial collection of mathematical records in general, Figshare serves as a crucial repository in particular for those requiring a system for the publication and citation of research figures and visual data, which are often not covered by most other repositories.

#### FindStat

FindStat is an online database dedicated to combinatorial statistics and their relations^43^. Inspired by the OEIS, the project was initiated in 2011 by Chris Berg and Christian Stump at the Université du Québec. Within the database, users can explore nearly 2000 combinatorial statistics, organized into 24 distinct categories, along with 296 maps and 24 collections. The repository is continuously updated with new entries that can be submitted through an online form. Each object receives a unique identifier for easy access to its properties page, explicitly mentioned in the metadata. Released under a CC BY 4.0 license, the data can be accessed in plain text or JSON format and integrates seamlessly with SageMath. FindStat fully complies with the FAIR principle of findability and partially complies with the remaining principles.

##### Relevance of the repository to the mathematical research community

FindStat, predominantly centered on combinatorial statistics, extends its utility to a wide range of mathematical fields through its comprehensive catalog of statistical data, maps, and combinatorial collections. Additionally, the repository’s direct integration with computational environments like SageMath significantly amplifies its value for the mathematical research community.

#### HAL open science

HAL open science (Hyper Article en Ligne) is an open access data repository for all academic fields. It is operated by the French data center *Centre pour la communication scientifique directe* (CCSD), which is part of the *French National Centre for Scientific Research* (CNRS). HAL was launched in 2001^44^ and stores around three million records. Since HAL is a major french research data infrastructure, many publications are written in French. Out of this, more than 130,000 entries are connected to mathematics as of June 2023. The majority of the mathematical entries are journal articles, conference papers or preprints. HAL stores approximately 3000 entries of non-written mathematical research data such as videos, software and photos. HAL offers generation of DOIs for hosted content, and various Creative Commons licenses are available. The HAL repository fully fulfills the FAIR principles, except for some reusability sub-principles.

##### Relevance of the repository to the mathematical research community

HAL open science is a general repository hosting a significant amount of mathematical research data. While the focus is the French-speaking mathematical community, an English interface is available as well.

#### Harvard Dataverse

The Harvard Dataverse is a cross-disciplinary institutional repository open for submissions from around the world. It runs on the open-source software Dataverse which has been in operation since 2006^45^ and is maintained by the Institute for Quantitative Social Science at Harvard University. The Harvard Repository is one of about 100 installations of the Dataverse software, and as of June 2023, it hosts about 170,000 records (6,000 Dataverse collections and 164,000 datasets). A Dataverse collection is a customizable collection of datasets (or a virtual repository) for organizing, managing, and showcasing datasets, with features allowing custom metadata and searchable metadata facet selection. It also contains meta-data about research items harvested from partner repositories. Overall, this repository contains about 1,7 million files. About 500 datasets and 120 Dataverse collections were tagged under ”Mathematical Sciences”. Multiple tags are possible for a given record, and “Computer and Information Science” as well as “Social Sciences” are most frequently associated with mathematical content. The data can be put under various licenses, including Creative Commons licenses and the Creative Commons Zero (CC0) waiver. The Harvard Dataverse meets most FAIR criteria.

##### Relevance of the repository to the mathematical research community

With a focus on members of Harvard university, this multi-discipline repository provides a significant number of datasets and research items related to mathematical sciences that might not be found anywhere else.

#### Network Repository

The Network Repository is a cross-disciplinary repository for network graph data^46^. Established in 2012, it has as of June 2023 about 6,600 networks classified in more than 30 domains, which are all available under the terms of a Creative Commons Attribution Share-Alike License (version not specified). The repository assigns a unique string identifier to each network and enables comparisons between different networks based on a given list of properties. A key feature of the site is that it offers interactive visualizations to explore the data. Users are invited to upload suitable graph data. The metadata related to each network is not available in a machine-readable format, and thus the repository does not fulfill the interoperability FAIR principles. The rest of the principles are partially fulfilled.

##### Relevance of the repository to the mathematical research community

The Network Repository provides essential data for mathematicians specializing in network theory and graph analysis.

#### On-Line Encyclopedia of Integer Sequences

The On-Line Encyclopedia of Integer Sequences (OEIS)^47^ was founded in 1964 and contains integer sequences and further information about the individual items. In 1996, the corresponding website was launched. Target groups are professional as well as amateur mathematicians. One of the key features is its ability to search and compare sequences. Each sequence is identified by a serial number, which makes it unequivocally identifiable. The information provided by the encyclopedia includes the sequence itself, paper references, links to material concerning a sequence, the formula used to generate it, keywords, as well as code in several programming languages and visualizations. It presently contains more than 300,000 sequences. The data contained in the Encyclopedia are made available under the CC BY-NC 4.0 license. The OEIS fulfills most of the FAIR criteria.

##### Relevance of the repository to the mathematical research community

The OEIS is an indispensable tool for mathematicians who are engaged in sequence-related research, offering a comprehensive and searchable database of integer sequences crucial for discoveries in various mathematical domains.

#### Open Science Framework

The Open Science Framework (OSF)^48^ is an open-source platform designed to support the entire research (-project) lifecycles. It offers functionalities to design studies, collect and analyze data as well as to publish reports and archive results. The platform is developed and maintained by the Center of Open Science (COS), a non-profit technology organization founded in 2013 that supports scientific research communities. Initially conceived for the field of psychology research, it has since its foundation become multidisciplinary. As of June 2023, it contains more than 4,500 files, 1,800 preprints and 1,300 projects in the field of mathematics. These represent only a minority among the over a million indexed preprints and over six million files hosted on the platform. For each project created using the platform, a DOI can be generated, and a license can be chosen, including Creative Commons, MIT, Apache, and GNU General Public licenses. The platform also offers file storage, version control and integration to citation management and storage tools, including Mendeley, Zotero, Figshare and GitHub. The platform adheres only partially to the FAIR principles, as it lacks persistent metadata, and does not include qualified references or detailed data provenance.

##### Relevance of the repository to the mathematical research community

While multidisciplinary, the Open Science Framework is becoming increasingly significant for the mathematical community, especially for those involved in data-driven research.

#### Open Science Library

The https://codeocean.com/explore?query=Mathematics&filter=all&refine=fieldOpen Science Library is part of the Code Ocean platform, which provides cloud-based computational environments. This allows computational researchers to share their data and the necessary code to enable others to reproduce the published analysis. It is run by the commercial company Code Ocean Inc. since 2016. The key feature of this platform is that all-needed components, i.e., data, source codes, and the runtime-environment are packaged together as a container ("compute capsule”). These containers are hosted on the platform and can be run from a web browser or locally without the need to install libraries or runtime-special environments. The platform contains more than 3000 capsules categorized into multiple research disciplines, including mathematics. A capsule is assigned a DOI, is built on FAIR principles, and allows easy migration across operating systems and platforms. Licenses vary and can be chosen by the authors (e.g. MIT, CC0, etc.).

##### Relevance of the repository to the mathematical research community

The Open Science Library, through Code Ocean, is particularly relevant for mathematicians working on computational research and seeking reproducibility of results.

#### Papers with Code

Papers with Code is an online resource that connects scientific papers with code implementations, datasets, methods, and evaluation tables. The portal offers additional valuable resources such as benchmarks that facilitate the comparison between state-of-the-art models. The entries overarching system can be explored through six specialized portals, which include the fields of machine learning, computer science, physics, astronomy, mathematics, and statistics. The specific portal for mathematics contains as of June 2023 more than 4000 datasets. Open to contributions from all users, the website operates under a CC BY-SA license. Each paper is assigned a string ID based on its title. This ID can be used to access the paper metadata through a REST API. The API also allows access to metadata regarding authors, conferences, datasets, evaluations, methods, models, and repositories. The portal fully adheres to the FAIR principles.

##### Relevance of the repository to the mathematical research community

Papers with Code is particularly vital for the applied mathematical community, especially for those concentrating on applied mathematics and computational methodologies, as it bridges the gap between theoretical research and practical implementation by providing direct access to code and datasets associated with scholarly papers.

#### *π*-base

https://topology.pi-base.org/*π*-base is a community database that focuses on topological counterexamples. Launched in 2014 by James Dabbs, the project has grown to include 79 spaces, each offering information through 146 properties and 344 theorems. To facilitate easy access, each object within the database is assigned a unique ID, allowing users to retrieve specific objects via their corresponding URLs. While the data is also available in a GitHub repository^49^, it lacks direct accessibility in a machine-readable format. The GitHub repository operates under a CC BY 4.0 license, but this licensing information is not explicitly stated on the website. Additionally, the repository features a guide outlining contribution conventions, offering users guidance on how to contribute effectively.

##### Relevance of the repository to the mathematical research community

-*π*-base is a valuable resource for mathematicians interested in topology, providing a wide array of topological counterexamples and associated data.

#### polyDB

polyDB is a database of discrete geometric objects which was launched in 2013 by Andreas Paffenholz and Silke Horn as an extension of the software package polymake^50^. As of June 2023 the database contains 21 collections that are classified into four groups: Manifolds, Matroids, Polytopes and Tropical Objects. In total, these collections contain more than 500 million documents. The data for each document and collection is stored as plain JSON and can be accessed through a REST API. To this purpose, both the collections and the documents receive a unique ID that can be used to access the data. Instructions to submit new collections are provided but there is no explicit information on the license under which the data is released. The repository partially fulfills all FAIR principles except the ones related to reusability.

##### Relevance of the repository to the mathematical research community

PolyDB is highly relevant to mathematicians working in the field of discrete geometry, offering extensive datasets on discrete geometric objects crucial for geometric and computational research.

#### Science Data Bank

The Science Data Bank (ScienceDB)^51^ is a public multidisciplinary research data repository for eScience which was launched in 2015. ScienceDB aims to become a long-term data sharing and data publishing repository in China that covers the entire spectrum of scientific fields. As of June 2023, it has close to 6 million open datasets, with over 25,000 being related to mathematics. These mathematics-related records consist of journal publications as well as datasets, slides, code data, and other multimedia content and cover a wide range of mathematical topics. Users have the option to select from a range of licenses, including CC-licenses, for licensing their published data. The uploaded data undergoes a review process by the curators and can be assigned a DOI. To facilitate access and utilization, ScienceDB provides an open REST-based API that allows users to retrieve metadata, conduct entry searches, and obtain dataset metrics. While ScienceDB aligns with the majority of the FAIR principles, it does not include qualified references in the metadata.

##### Relevance of the repository to the mathematical research community

ScienceDB is a multi-disciplinary repository with a large number of mathematics-related entries in particular from Chinese researchers that might not be found in other repositories.

#### SuiteSparse Matrix Collection

The SuiteSparse Matrix Collection^52^ is a curated set of sparse matrices that arise in real applications from a wide spectrum of domains, such as thermodynamics, material science and optimization. The target group is the numerical linear algebra community, which is provided with curated data allowing for robust and repeatable experiments or for benchmarking purposes. Matrices are identifiable by ID and related metadata, such as the matrix norm or the structural rank. The matrices can be accessed over several interfaces for Java, Matlab, Julia and Python and are made available under a CC BY 4.0 License. The SuiteSparse Matrix Collection fulfills less than half of the FAIR criteria.

##### Relevance of the repository to the mathematical research community

The SuiteSparse Matrix Collection is of importance to mathematicians involved in numerical linear algebra and optimization problems.

#### The House of Graphs

The House of Graphs (HoG) provides a searchable database of graphs and network structures. It was created in 2013 and includes a growing collection of graphs with nearly 22,000 entries^53^ that are classified based on various characteristics, such as size, degree distribution, and connectivity. Registered users can add new graphs to the database and existing graphs can be downloaded in various formats, along with their corresponding metadata. The HoG also provides tools for graph visualization, enabling researchers to gain insights into the structures of the graphs in the database. No information about the used licenses is given. The current implementation of the repository adheres to most of the FAIR principles for findability and accessibility but does not comply to the principles for interoperability and reusability.

##### Relevance of the repository to the mathematical research community

The House of Graphs is an essential resource for mathematicians with a focus in graph theory and network sciences.

#### Wikidata

Wikidata is an open, cross-disciplinary and multilingual collaborative knowledgebase^54^ that has taken Wikipedia’s “anyone can edit” approach to the Linked Open Data world. It is built on MediaWiki, with a set of extensions for handling of mathematical expressions^55,56^ and structured data, collectively known as Wikibase. Launched in 2012, Wikidata currently contains about 1.5 billion statements about 100 million items, and 1 million lexemes, expressed via about 10,000 properties. The over 20,000 monthly contributors have made a total of about 2 billion edits so far, mostly via semi-automated tools. The data is licensed CC0 and accessible as dumps, via APIs, via a SPARQL endpoint, and via a range of tools for browsing or editing. Wikidata meets all FAIR criteria^57^. Roughly 1‰ of the content is math-related, including math publications, mathematicians, mathematical research organizations, societies, databases, conferences, software packages, algorithms, theorems, proofs, numbers, number series, and more, albeit usually with incomplete coverage^58,59^. Wikidata’s 2022 growth rate was approximately 4% for items, 12% for properties, 46% for lexemes and 6% for statements.

##### Relevance of the repository to the mathematical research community

Wikidata supports the mathematical research community by offering structured data about mathematical concepts, mathematicians, and publications, facilitating easy access and cross-referencing.

#### Zenodo

Zenodo^60^ is an open science data repository maintained by CERN based on the open-source Invenio framework. It was created in 2015 to provide a solution for scientists to store, share, and publish their research data and digital artifacts, such as research papers, software, or data sets. The Zenodo system provides users with a range of services, including long-term data preservation, versioning, data citations, and DOIs. The repository has a simple and user-friendly interface, making it easy to upload and manage research data. Zenodo is integrated with a range of other systems and services, including Github, CERN’s Open Data Portal, and the European Open Science Cloud, among others. The Zenodo repository contains almost 3 million records, with the majority of them being freely accessible.

##### Relevance of the repository to the mathematical research community

Zenodo offers a large number of math-related research objects, and stands out in particular for those working with extensive datasets or large computational projects, as it provides up to 50 GB of free storage per item.

## Wiki-based Math Data Sharing

Mathematical research data is frequently published online through wiki-based systems. These allow for the rapid creation and iterative editing of entries in a collaborative environment. Due to the convenience of this approach, there are several systems dedicated to specific mathematical disciplines. Table 6 presents a selection of exemplary systems that fall into this category.

The table includes a collection of systems, all of which operate on MediaWiki, except for nLab and MathRepo. nLab is built on Instiki, a wiki software based on Ruby on Rails, and MathRepo (Mathematical Research Data Repository) is based on GitLab. Among the systems listed, there are a few noteworthy examples worth highlighting: the Encyclopedia of Math, Complexity Zoo, MathRepo, and nLab. The Encyclopedia of Math is an online wiki initially created by the Springer Verlag and managed in cooperation with the European Mathematical Society. It hosts over 8,000 articles covering advanced mathematical topics, which can be updated by users and undergo editorial board review for accuracy. The Complexity Zoo, initiated by Scott Aaronson in 2002, aims to catalog all classes of computational complexity and currently documents over 500 complexity classes. MathRepo is a repository for mathematical research data of and by the Max Planck Institute for Mathematics in Sciences (MPI-MiS). It has been operational since 2017 and as of 2023 contains more than 70 records. Lastly, nLab is a collaborative system that includes more than 18,000 pages spanning mathematics, physics, and philosophy, with a strong emphasis on type theory, category theory, and homotopy theory.

While this approach facilitates the sharing of research data, the existing implementations currently show limited adherence to the FAIR principles. Merely relying on the default environment offered by a MediaWiki instance does not inherently ensure satisfactory compliance with FAIR principles. Achieving proper FAIR adherence requires a deliberate effort and the addition of supplementary measures.

Most of the systems featured in this table do not assign persistent identifiers to their resources. Instead, the resources are solely accessible through URLs, without any guarantee of persistence. Despite the availability of MediaWiki’s API for accessing metadata on existing MediaWiki sites, the listed systems do not use this mechanism to provide comprehensive metadata describing their stored resources. Qualified references to other resources are rarely provided, and explicit license information is seldom included. Only in cases where user pages are available for each user, some individuals may voluntarily add identifiers, such as an ORCID ID, which can serve as qualified references. However, this information is not directly included in the retrieved metadata.

In summary, using MediaWiki as the foundation for a research data systems can serve as a valuable initial step in establishing a collaborative environment for resource sharing among users. However, to ensure adherence to the FAIR principles, additional features must be implemented beyond the basic configuration. These include assigning persistent identifiers to resources, making these identifiers readily available along with comprehensive and contextual metadata through the provided API, implementing controlled vocabularies, including qualified references to other resources, and clearly publishing license information.

## Discussion

The previous section has introduced several prominent systems for sharing mathematical research data, highlighting their distinctive characteristics. By examining the collected data for each repository, a comparison can be made in terms of their focus, size, and available features.

An important aspect to take into account is the system’s capability to assign persistent identifiers to its resources. This includes both the items stored within the system and the references to other resources, such as authors and publications. This ability directly impacts the indexing and citation process of resources within the system, and it is also closely tied to the FAIR principles, particularly in terms of facilitating findability.

Open data systems can also be compared based on their metadata management features. In this context, it is crucial to assess whether a clear distinction is made between the data itself and the accompanying metadata. Additionally, it is important to determine whether the metadata includes relevant fields that describe the resource, such as qualified references to other resources in the form of persistent IDs. For mathematical resources, relevant metadata fields may include but are not limited to: title, author(s), abstract, keywords, mathematical subject classification codes (MSC), mathematical formulae, theorem statements, proofs, datasets used or generated, software tools or algorithms employed, publication status (e.g., preprint, published), and associated arXiv or DOI identifiers (similar to the *Dublin Core Metadata Element Set*). Another important feature to consider is the inclusion of timestamping data within the metadata, which can indicate when the resource was created or updated. Also relevant to the management of metadata is the provision of API endpoints for retrieving metadata, search capabilities, and submission functionalities. These features are directly related to the FAIR principle of interoperability. Lastly, we have also analyzed the status of the current systems with regard to the reusability of their data.

This assessment is based on their adherence to the FAIR principles, particularly focusing on aspects such as licensing information availability (including licensing terms) which is integral to ensuring data can be reused effectively.

The diverse range of features and services provided by repositories plays a crucial role in promoting open science practices and fostering collaborations among researchers. Given the importance of online data repositories in scientific research and their variations in focus, functionality and FAIR compliance, researchers should carefully select the most suitable repository based on their research needs and the nature of the data they intend to share.

Based on all the evaluated factors, we present a thorough analysis of the current status of open systems in the field of mathematics, which can support this selection process, including a specific analysis of the current status of FAIR compliance.

### The Status of Open Data Systems

The growing number of open data systems in science and academia indicates a significant shift toward the democratization of knowledge and the promotion of open science. These systems provide the necessary infrastructure for storing, sharing, and reusing data, which promotes collaboration, transparency, and the advancement of research. Tables 3 and 4 summarize our evaluation of the current status of these systems and their usage, especially within mathematical research.

The focus of a system plays a fundamental role in determining the features it offers. This is particularly important in relation to the discipline or type of data stored. As can be observed from the second column in Table 3, systems can be categorized into two distinct types: *multidisciplinary* (containing a significant amount of math data) and *specialized* (exclusively storing mathematical data). The respective focus determines the type of data a system accepts and directly impacts decisions regarding the technology used, the need for curation and review processes, the implementation of a metadata scheme, and the policies for persistent data storage.

Within the multidisciplinary systems, there is a subgroup that consists of general-purpose data systems such as 4TU research data, Figshare, Harvard Dataverse, the Science Data Bank, and Zenodo. These aim to facilitate the sharing of research data and are therefore targeted at a similar audience. Consequently, it is not surprising that some of these systems are partially built on the same software, as is the case with 4TU Research data, which is built on Figshare. As a result, these systems offer a similar range of features, including the assignment of DOIs to resources, ensuring long-term data preservation, providing options for both public and private repositories, and timestamping metadata for each uploaded version.

Another group of multidisciplinary systems focuses on enhancing collaboration, study design, and data analysis among researchers. One example is the Open Science Framework, which serves as a general-purpose data repository but also offers functionalities to manage the entire research project lifecycle. Similarly, the Open Science Library falls into this category. Although the Open Science Library is limited to storing data and source code packaged as computational capsules, it provides researchers with tools for study design, data collection and analysis, report dissemination, collaboration, and integration with other services.

Lastly, there is a third group of multidisciplinary systems that exclusively house textual data in the form of scientific publications or metadata associated with them. Examples of such systems include arXiv, HAL, and Papers with code. arXiv and HAL primarily focus on storing scientific articles, while Papers with code goes a step further by linking these articles to code repositories and available benchmarks.

On the opposite end of the spectrum, we encounter a group of systems that specialize in storing specific types of data. Examples include Archive of Formal Proofs, the Database of Ring Theory, the House of Graphs, and the On-Line Encyclopedia of Integer Sequences, which focus on storing proofs, ring data, graph data, and integer sequences, respectively. Typically, these are built using customized code based on available frameworks. Many of these systems also choose to open-source their source code and data on platforms like GitHub or GitLab. This approach offers several advantages for systems with a relatively small number of items, such as *π*-base or the Database of Ring Theory. It provides redundancy for data storage, facilitates authentication mechanisms, and enables users to submit new content through pull requests.

Another key aspect that varies among the evaluated systems is how they manage persistent identifiers. This includes the assignment of identifiers to stored resources, as well as the inclusion of qualified references to other resources like authors and publications. While the majority of the considered systems assign persistent identifiers to their resources, there are some exceptions, such as Archive of Formal Proofs, the Network Repository, and Papers with Code which can only refer to resources using a URL, without guaranteeing persistence. In contrast, the remaining systems all provide internal persistent IDs for their resources. Many general-purpose systems, in addition to their internal IDs such as arXiv ID, idHAL, or Zenodo ID, also assign a DOI. The allocation of a DOI is particularly important as it simplifies citation practices and helps to monitor the use of data, as well as giving credit to data providers.

None of the evaluated systems enforces an assignment of a persistent identifier to the resource creator. However, some systems offer optional fields to include identifiers, such as the ORCID ID. Notably, this functionality is primarily supported in multidisciplinary systems and not in any of the math-specific portals. Additionally, some of the general-purpose systems generate internal author IDs on an optional basis, as seen in arXiv with the arXiv author ID. While some systems only store author names as plain text, others like OSL or polyDB also include affiliation information. Certain systems enable the creation of profile pages for individual authors, allowing them to voluntarily add identifiers. This feature is present in systems based on MediaWiki, including Wikidata and OEIS.

In contrast, when it comes to referencing publications, the support for persistent identifiers is more prevalent compared to authors, as Table 4 confirms. Nearly all systems employ some form of identifier to reference other publications. The most common method is through the use of DOIs. However, there is an interesting exception in the case of FindStat, which uses MathSciNet IDs to reference the mentioned publications. Furthermore, cross-referencing based on internal identifiers from other systems is also present. For example, both FindStat and the Encyclopedia of Graphs include references to integer sequences using the OEIS ID.

Another distinguishing factor is the approach to data review and curation. Specialized repositories focused on specific domains prioritize data curation to ensure the publication of high-quality data. Due to their specialization, these repositories store limited types of data, making the data curation process more manageable. In contrast, general-purpose repositories that cover a wide range of disciplines and types of data often do not perform data curation or review. These repositories typically conduct some form of monitoring for uploaded datasets to ensure compliance with site policies. In some cases, they only review the uploaded metadata, as seen in the case of 4TU Research Data. Other repositories, such as Harvard Dataverse, review all deposits to ensure reusable data are included, offer free consultation services to help users set up their collections, ensure proper metadata, and offer data curation as a paid service. In cases where only basic data control is implemented, it is also common practice to include timestamp information whenever the uploaded data is updated or modified.

The availability of API endpoints for (meta)data retrieval varies among the evaluated systems. Notably, more than a third of them do not offer any API functionality, limiting access to their data solely through a web browser. This absence often leads to a lack of clear differentiation between data and metadata, significantly hindering data reusability. However, among the systems that provide API endpoints for resource exploration and search, around half of them also support the submission of new resources through the API. It is worth noting that these capabilities are predominantly found in general-purpose repositories.

The licensing terms for the included systems vary considerably. Numerous systems provide versatile licensing options, such as numerous Creative Commons (CC) licenses, BSD, LGPL, MIT, GPL, Apache, and copyright licenses. In some cases, a default license is suggested that can be changed by the author. For example, both 4TU Research Data and Harvard Dataverse default to CC0 for data release. Conversely, Biomodels and Wikidata strictly require the use of CC0.

Judging by the quantity of items (i.e. datasets, papers, proofs, models, sequences, etc.) available, arXiv, Figshare, HAL, Zenodo, and the Open Science Framework are most popular within the academic community among the multidisciplinary systems. Other specialized systems, however, store an even larger number of items, such as Wikidata, the Encyclopedia of Graphs or PolyDB, with millions of items each.

In conclusion, our analysis of the current landscape of open data systems reveals a growing utilization and recognition of repositories, platforms, and portals for sharing research data within the scientific communities in general, and in the context of mathematics in particular. The establishment of dedicated repositories for mathematical data, such as the Archive of Formal Proofs and the On-Line Encyclopedia of Integer Sequences, the evident increase in mathematical datasets hosted in multidisciplinary repositories like Zenodo and Figshare, and the heightened awareness and adoption of the FAIR principles among researchers and institutions underline this trend. However, our analysis also identifies unresolved challenges that need addressing to fully leverage the potential of these systems. These include the need for wider adoption of persistent identifiers to enhance the citability of datasets, the harmonization of licensing practices to facilitate the sharing and reuse of data, and the improvement of interoperability features, particularly through the development of more comprehensive APIs. As the data landscape continues to evolve, these systems must adapt to meet the changing needs of researchers and ensure the continued advancement of open science. In the next section, we will specifically examine the status of FAIR compliance among the evaluated systems.

### FAIR compliance

Table 5 provides an assessment of the systems included in this work with regard to their adherence to the FAIR principles. The data gathered in this table reveals two main points. Firstly, it highlights the shortcomings regarding FAIR compliance within the current ecosystem, specifically within the domain of mathematics. Secondly, it brings to attention the challenges encountered in adhering to certain FAIR principles, which are often not only technical but arise also from ambiguous interpretations of the principles and due to the absence of relevant standards within certain disciplines.

**Table 5:** FAIR compliance of included portals, sorted alphabetically.

Portal Name	Findable				Accessible				Interoperable			Reusable			
	F1	F2	F3	F4	A1	A1.1	A1.2	A2	I1	I2	I3	R1	R1.1	R1.2	R1.3
4TU Research Data	✓	✓	✓	✓	✓	✓	✓	✓	✓	✓	✓	✓	✓	✗	✗
Archive of Formal Proofs	✗	✓	✗	✓	✓	✓	✓	✗	*✓*	✗	✗	✗	✓	✓	✓
arXiv	✓	✓	✓	✓	✓	✓	✓	✓	✓	✓	✗	✓	✓	✗	✓
Biomodels	✓	✓	✓	✓	✓	✓	✓	✓	✓	✓	✓	✓	✓	✓	✓
Database of Ring Theory	✗	✓	✗	✓	✓	✓	✓	✗	✗	✗	✗	✗	✗	✗	✓
Encyclopedia of Graphs	✓	✓	✗	✓	✓	✓	✓	✗	✗	✗	✗	✗	✓	✗	✓
Figshare	✓	✓	✓	✓	✓	✓	✓	✓	✓	✓	✓	✓	✓	✗	✗
FindStat	✓	✓	✓	✓	✓	✓	✓	✗	✓	✓	✗	✗	✓	✗	✓
HAL	✓	✓	✓	✓	✓	✓	✓	✓	✓	✓	*✓*	✓	✓	✗	✓
Harvard Dataverse	✓	✓	✓	✓	✓	✓	✓	✓	✓	✓	*✓*	✓	✓	✗	✗
Network Repository	✓	✓	✗	✓	✓	✓	✓	✗	✗	✗	✗	✗	✓	✗	✗
OEIS	✓	✓	✗	✓	✓	✓	✓	✗	✓	✗	✓	✓	✗	✓	✓
Open Science Framework	✓	✓	✓	✓	✓	✓	✓	✗	✓	✗	✗	✓	✓	✗	✗
Open Science Library	✓	✓	✗	✓	✓	✓	✓	✓	✓	✓	✓	✓	✓	✗	✗
Papers with Code	✓	✓	✓	✓	✓	✓	✓	✓	✓	✓	✓	✓	✓	✗	✓
*π*-Base	✓	✓	✗	✓	✓	✓	✓	✗	✗	✗	✓	✗	✗	✗	✓
polyDB	✓	✓	✗	✓	✓	✓	✓	✗	✓	✓	✗	✗	✗	✗	✓
Science Data Bank	✓	✓	✓	✓	✓	✓	✓	✓	✓	✓	✗	✓	✓	✗	✗
SuiteSparse Matrix Collection	✓	✓	✗	✓	✓	✓	✓	✗	✗	✗	✗	✗	✓	✗	✗
The House of Graphs	✓	✓	✗	✓	✓	✓	✓	✗	✗	✗	✗	✗	✗	✗	✓
Wikidata	✓	✓	✓	✓	✓	✓	✓	✓	✓	✓	✓	✓	✓	✓	✓
Zenodo	✓	✓	✓	✓	✓	✓	✓	✓	✓	✓	✓	✓	✓	✗	✗

**Table 6:** Mathematical data sharing systems based on MediaWiki (except nLab and MathRepo).

System	Math Focus	URL
Boolean Zoo	Boolean analysis	booleanzoo.weizmann.ac.il
Complexity Zoo	Complexity classes	complexityzoo.net
Encyclopedia of Math	Articles in the field of mathematics	encyclopediaofmath.org
GroupProps	Group properties	groupprops.subwiki.org
MathRepo	Research items from the MPI-MiS	mathrepo.mis.mpg.de
MOR Wiki	Model benchmarks	morwiki.mpi-magdeburg.mpg.de
nLab	Category theory	ncatlab.org
The Knot Atlas	Knots	katlas.org
The Manifold Atlas	Manifolds	map.mpim-bonn.mpg.de

In relation to the findability principles, almost all investigated systems assign unique and persistent identifiers to their resources, thereby satisfying principle F1. Moreover, almost all of the systems provide rich metadata on each object, thereby also fulfilling principle F2. It is worth noting, however, that while most systems do establish a clear distinction between data and metadata, half of them do not explicitly reference the persistent identifier within the metadata, resulting in non-compliance with principle F3. This principle is also immediately not fulfilled by those systems that do not assign persistent identifiers in the first place. Finally, the adherence to principle F4 concerning the indexing of the resources does not pose a technical challenge and is fulfilled by all systems.

Given that this article focuses on open systems, all examined systems adhere to principle A1 regarding accessibility, as well as its corresponding sub-principles. However, it is worth mentioning that only half of these comply with principle A2, which guarantees the availability of metadata even if the original data resource is deleted. The non-adherence to this principle can often be attributed to the absence of a clear distinction between data and metadata, coupled sometimes with the lack of persistent identifiers.

Adhering to the interoperability principles requires the publication of metadata in a format that can be readily interpreted by machines. In this regard, approximately two-thirds of the systems grant access to metadata via an API, but only less than half of them employ controlled vocabularies for metadata description. Furthermore, qualified references to other metadata, such as DOIs for publications or ORCID IDs for referenced authors, are included in only half of the systems, as can also be seen in Table 4.

With regard to the principles concerning reusability, principle R1 requires enriching metadata to assess the usefulness of data within a given context. While a clear definition of rich metadata is lacking, our evaluation has determined that only half of the systems offer some form of contextual metadata. Moreover, six of the included systems do not disclose metadata with explicit license information, thereby failing to meet the requirements for principle R1.1. Adhering to principle R1.2 presents a technical challenge due to the difficulties involved in describing data provenance and workflows by means of controlled vocabularies and machine-readable formats. This principle is easily fulfilled by systems that exclusively store specific data types, such as descriptions of particular mathematical objects, but poses a challenge in multidisciplinary systems capable of storing diverse data resources. For the same reason, compliance with principle R1.3, which mandates conformity to domain-relevant community standards, proves challenging for multidisciplinary systems. The adherence to this principle relies on the existence of community standards, which may not always be present, particularly in niche disciplines, consequently impeding its compliance.

A general overview of these results show that, with the exception of principles F3 and A2, most of the systems fulfill the FAIR principles for findability and accessibility. However, compliance rates are comparatively lower in the areas of interoperability and reusability. In particular:

1. Findability principles do not present a technical challenge and are mostly fulfilled by all systems. The main shortcoming in the mathematical ecosystem is the absence of explicit references to persistent identifiers (F1) in the metadata, thereby preventing the fulfillment of principle F3.
2. Accessibility principles are entirely fulfilled, except for principle A2. This last principle requires a clear distinction between data and metadata, along with ensuring the availability of metadata even if the original resource is deleted.
3. The compliance of the interoperability principles is coupled with the technical challenges of publishing rich metadata in a machine-readable format. Even among the systems that fulfill principle I1, there is still a lack of compliance regarding the use of controlled vocabularies with persistent identifiers and the inclusion of qualified references to other metadata.
4. The reusability principles are only partially fulfilled among the covered systems. The compliance with these principles could be improved by adding contextual metadata, explicit license information and metadata on data provenance. The adherence to principle R1.3 requires not only a technical implementation but also the definition and acceptance of well-defined community standards within each discipline, and in particular, in the different mathematical disciplines.

### Requirements for Open Data Systems

While conducting our analysis of open data systems, with a primary focus on mathematical research, we have not only catalogued existing features and practices but also identified critical gaps and areas for improvement. This has led us to formulate the following set of foundational requirements that open data systems must meet to effectively support the research community as a whole, with particular enhancements for the mathematical community. These requirements emerge naturally from our analysis, and address the *technical*, *user-centric*, and *legal* dimensions crucial for the management, sharing, and preservation of research data. They provide a summary of the lessons learned from existing systems and a path forward for the development and enhancement of future platforms. In presenting these requirements, we aim to contribute to the broader discourse on open science by highlighting how systems can evolve to meet the specific needs of researchers, ensure data integrity and accessibility, and promote ethical practices. Where relevant, we have pointed out the specific significance of these requirements for the mathematical community, highlighting the unique aspects within the broader scientific ecosystem.

### Technical requirements

#### Security

Robust security protocols are imperative to safeguard data against unauthorized access or alterations. Implementing stringent security measures such as encryption, access control, and audit trails is non-negotiable, protecting the integrity of mathematical research data.

1. Scalability and Reliability: The system must be able to scale in order to accommodate growing data volumes and user requests, ensuring high availability and robust data recovery processes. This is especially crucial for mathematical research areas that generate, store, and analyze extensive datasets
2. Interoperability: The system must utilize standard data formats and protocols to ensure that the data can be readily accessed and utilized by multiple systems and applications. It should also facilitate the integration and linking of data from various sources. This is crucial for mathematical platforms due to the diverse tools and software used in mathematics. Standardized data formats enable seamless integration with mathematical software, computational notebooks, and other data repositories.
3. Findability: Effective data indexing and advanced search capabilities are essential for assisting users in locating the required data. This entails offering metadata, unique identifiers, and a robust search engine. Advanced data indexing and search functionalities are vital for navigating the vast and varied landscape of mathematical data (see Table 1). This complexity demands sophisticated search mechanisms beyond basic keyword searches, aiding researchers in efficiently discovering the exact data they require.
4. Security: The system must provide robust security measures to prevent unauthorized data access or modification. This includes encryption, access control, and auditing functions.

### User-centric requirements

1. Usability: The system’s interface should be simple and intuitive. Users should be able to upload, retrieve, and manipulate data without difficulty. This is essential to accommodate the diverse technical backgrounds in particular in the mathematical community, from pure mathematicians to applied researchers and educators on various levels.
2. Accessibility: All data should be accessible to users with varying requirements and resources, including those with disabilities. This is critical in particular in mathematics, a field where contributions can come from any part of the world, often with minimal resources - unlike in other disciplines requiring expensive lab equipment. Moreover, offering programmatic access via APIs is essential for integrating these datasets into broader research workflows.
3. Support: Users should have access to documentation, tutorials, and user support in order to understand how to use the system and its data. While universally important, in the context of mathematics, and particularly in resource-limited settings, access to comprehensive support and documentation can empower researchers to fully utilize data systems with limited external assistance.

### Legal and Ethical Requirements

1. Licensing and Citability: The system should provide explicit licensing information for each dataset, indicating how it may be utilized and whether or not it should be cited.
2. Compliance with FAIR principles: The data must be discoverable, accessible, interoperable, and reusable (FAIR). This entails making data readily discoverable, accessible under open licenses, compatible with other data sets, and well-documented so that it can be reused in a variety of contexts.
3. Privacy and Ethics: If the system hosts personal data, it must comply with privacy regulations such as the General Data Protection Regulation (GDPR). Establishing and adhering to ethical guidelines for the collection, use, and sharing of data is necessary.
4. Transparency and Accountability: The system should have transparent data collection, utilization, and governance policies and be responsible for their enforcement.

### Challenges and obstacles for Open Data Systems

Implementing open data systems that are widely utilized is a challenging task, complicated with numerous challenges and obstacles. While the need for these systems is widely acknowledged, a number of significant obstacles remain. In the following, we list some of these multifaceted obstacles, underscoring not only the broad issues that are relevant to all fields but also pointing out the distinct challenges inherent in the mathematical sciences. We also suggest some ideas towards resolving these barriers, highlighting the importance of specialized approaches alongside general strategies.

1. Data Privacy and Security: Ensuring data privacy and security is a significant challenge. It is a delicate task to balance the openness of data with the need to safeguard sensitive information. In Europe, laws such as GDPR have imposed stringent data management requirements, making it even more difficult for open data systems to comply. While sensitive information may not be the first concern that comes to mind in mathematical research compared to medical or social sciences data, issues around data privacy and security can still arise. For example, large datasets might include data derived from collaborative research with industry partners or shared computational models could reveal proprietary or sensitive strategies.
  *Solutions Approach:* Automated data anonymization techniques could be employed, allowing sensitive information to be obscured with only minimal manual intervention after uploading the data.
2. Data Standardization and Interoperability: The absence of data standardization is a significant technical obstacle. It can be challenging to integrate disparate data sources onto a single systems due to the diverse formats of the data. This lack of interoperability can reduce the system’s utility, as users may have difficulty locating, comparing, and utilizing data from various sources. In mathematics, this challenge is particularly relevant due to the diverse nature of mathematical research. Unlike disciplines with more homogenous data types, mathematics would benefit greatly from standardized data formats that facilitate integration across subfields and software.
  *Solutions Approach:* Development and use of open-source tools and libraries that support open data standards should be encouraged. Collaboration with international societies and journals to endorse and disseminate these standards would accelerate their adoption.
3. Data integrity and Curation: Another challenge is ensuring the integrity of data. Inaccurate data can lead to erroneous conclusions, so open data systems must have robust data validation and curation procedures. Curation in this context involves ensuring the accuracy, integrity, and usefulness of data across its lifecycle. This encompasses validation checks to verify data correctness, metadata standards for enhanced findability and reusability, and review processes to maintain quality. While parts of this can be automated, this is a resource-intensive endeavor and equally important over all fields of science that frequently necessitates subject-matter specialists and substantial computational resources.
  *Solutions Approach:* Open data systems can implement automated tools for preliminary data checks and leverage community-driven peer review processes to maintain data quality. Establishing partnerships with academic institutions and leveraging crowdsourced expertise can also provide the necessary resources for thorough data curation without overwhelming system resources.
4. Funding and Sustainability: Open data systems necessitate substantial resources for development, maintenance, and enhancement. This includes technical infrastructure, personnel, and data curation on an ongoing basis. Obtaining stable, long-term funding for these activities is typically difficult, especially for systems that provide unrestricted access to their resources. This challenge can be particularly pressing in fields where the direct applicability of research and therefore its funding may be less obvious to external stakeholders, which potentially is the case in some branches of mathematics.
  *Solutions Approach:* Demonstrating the broader impact of these open data and open science systems on scientific discovery and education could attract support from sources such as government agencies, private foundations, and industry partners.
5. User Engagement and Training: Lastly, promoting the adoption and proper utilization of open data systems is a challenge in and of itself. Many potential users may lack the technical expertise required to utilize these systems effectively. This requires investments in user training and ongoing efforts to enhance usability and accessibility. In mathematics, the technical barrier to entry for utilizing open data systems can vary widely depending on the user’s background. For example, applied mathematicians may require different resources compared to theoretical mathematicians.
  *Solutions Approach:* Hosting workshops, and training sessions in combination with online communities or forums where users can share tips, ask questions, and provide feedback can foster user compentencies, a sense of ownership and collaboration among users.

In conclusion, the implementation of open data systems, especially within the mathematical community, presents a unique set of challenges that mirror those in other scientific fields but also include math-specific contexts that require tailored solutions. However, the potential benefits to the scientific community and the general public make it worthwhile to overcome these obstacles. This demands the concerted efforts of the entire scientific community, including researchers, technical experts, funders, and institutions, aimed at fostering an environment where the full potential of open data can be actualized.

### Lessons learned

In this article, we gave an overview and an evaluation of the current status of open data systems, with a focus on the field of mathematics. This analysis has yielded the following valuable insights, or *lessons learned*:

1. *The Importance of User-Friendly Platforms*: Our work highlighted the varied levels of complexity across data repositories, from highly technical platforms to more accessible ones. A notable example is the comparison between the *Archive of Formal Proofs* and platforms like *Zenodo*. The former requires a deep understanding of formal proofs and theorem provers, whereas Zenodo’s interface is designed to be intuitive for a broad user base. This contrast underlines the critical need for repositories to balance sophistication with accessibility to encourage widespread data sharing among mathematicians, even outside their own domain.
2. *Varied Data Standards Pose Challenges*: Our analysis of repositories like *polyDB* and the *On-Line Encyclopedia of Integer Sequences* revealed the wide range of data formats and standards within the mathematical community. This diversity, while reflective of the field’s richness, underscores the challenges of data interoperability and standardization. The successful integration of data from these repositories into broader analyses or across different platforms illustrates the necessity of developing and adhering to common data standards.
3. *Effective Metadata Enhances Data Utility*: Observations from our study, particularly the advanced metadata functionalities of for example *Harvard Dataverse* and *Wikidata*, demonstrate the significant impact of well-structured metadata on data discoverability and usability. These platforms offer sophisticated metadata schemas that facilitate precise searching and data retrieval, showcasing the direct correlation between comprehensive metadata provision and enhanced data utility.
4. *The Need for Continuous Data Management*: Insights into the dynamic nature of data were gained through examining repositories with robust version control and update mechanisms, such as the *Open Science Framework*. The evolution of datasets over time, due to corrections, enhancements, or expansions, necessitates ongoing data management to ensure the continued relevance and accuracy of the data shared.
5. *Funding and Sustainability are Key for Longevity*: The analysis revealed that the sustainability of open data systems often hinges on stable funding sources, as seen in the operation models of *arXiv* and *HAL*. These repositories have established mechanisms to ensure their longevity, highlighting the critical role of financial and institutional support in maintaining open data infrastructures.

Each of these points highlights the complexities and opportunities within the current ecosystem of data sharing in mathematics - and beyond. With this list, we aim to provide actionable insights that can inform future developments and strategies in the management and dissemination of (mathematical) research data. Despite the notable advancements in data sharing within the field of mathematics, as detailed in previous sections, there is still a pressing need for ongoing improvement and adherence to best practices. Moreover, enhancing these efforts requires authors to not only contribute to the transparency and reproducibility of scientific research but also to actively engage in advancing the collective knowledge within the mathematical community and beyond.

## Data Availability

No datasets were generated during this study.

## Code availability

No codes were generated during this study.
